# Validation of food variety and dietary diversity scores as indicators of micronutrient adequacy among pregnant women in the northern zone of Sidama, Ethiopia

**DOI:** 10.3389/fpubh.2025.1536419

**Published:** 2025-06-11

**Authors:** Kaleb Mayisso, Tafese Bosha, Dessalegn Tamiru

**Affiliations:** ^1^School of Nutrition, Food Science and Technology, Hawassa University, Awasa, Ethiopia; ^2^Departments of Nutrition and Dietetics, Faculty of Public Health, Jimma University, Jimma, Ethiopia

**Keywords:** dietary diversity score, Ethiopia, food variety score, micronutrient adequacy, pregnant women

## Abstract

**Background:**

A diet that includes a variety of foods provides all the essential nutrients needed to meet nutritional requirements. However, the relationship between dietary diversity and adequate micronutrient intake has not been consistently established across various cultural contexts. Notably, no studies have successfully validated a link between dietary diversity scores and nutrient adequacy in Ethiopia. Therefore, we aimed to validate food variety and dietary diversity scores as proxy indicators of nutrient adequacy among pregnant women in Ethiopia.

**Materials and methods:**

A multi-stage systematic random sampling method was used to select study participants from March 1 to March 30, 2024. The multiple-pass 24-h dietary recall method, incorporating the standard nine food groups, was employed to estimate the dietary diversity score of pregnant women. The nutrient adequacy ratio (NAR) was calculated based on the mean adequacy ratio of various micronutrients. Receiver operating characteristic (ROC) curve analysis was used to determine optimal cutoffs for dietary diversity and food variety scores by balancing sensitivity and specificity..

**Results:**

MDD-W exhibited a positive correlation (*ρ* = 0.159, 95% CI: 0.065–0.250) (*p* = 0.001) and demonstrated strong predictive ability (AUC = 0.839, 95% CI: 0.80–0.88; *p* value = 0.001) for the mean adequacy ratio in assessing micronutrient adequacy. The sensitivity and specificity of the MDD-W at the standard cutoff of ≥5 food groups were found to be 69.9 and 5.3%, respectively. Additionally, the optimal cutoff points for dietary diversity and food variety scores in predicting micronutrient adequacy were determined to be 3.42 and 4.67, respectively. The food variety indicator based on nine food groups showed a negative correlation (*ρ* = −0.402, 95% CI: −0.137-0.053) (*p* > 0.05) and remained a poor predictor (AUC = 0.709, 95% CI: 3.49 to 4.03) of the mean adequacy ratio.

**Conclusion:**

MDD-W was positively correlated with micronutrient intake adequacy and demonstrated good predictive ability. At the standard cutoff of ≥5 food groups, its sensitivity and specificity were 69.9 and 5.3%, respectively. However, the findings differed from those of other studies, and discrepancies with FAO recommendations regarding the cutoff values and performance levels of MDD-W were observed, indicating a need for further investigation.

## Introduction

Pregnancy is a unique period in a woman’s life, marked by anatomical, physiological, and biochemical changes affecting nearly all her organ systems (1). During this time, increased energy, food, and nutrient intake are essential to support the needs of both the mother and the developing fetus (2). Incorporating whole grains and healthy fats can further enhance overall health, ensuring that both the mother and the fetus receive the nutrients essential for optimal growth and development. It is also crucial to consult healthcare providers to tailor dietary choices to individual needs throughout pregnancy (3, 4).

However, only 28.8% of pregnant women worldwide meet the Minimum Dietary Diversity for Women (MDD-W) (5). In resource-poor settings, women of reproductive age often experience inadequate micronutrient intake due to diets dominated by starchy staples, with minimal or no inclusion of animal products, fresh fruits, or vegetables (3). Insufficient maternal micronutrient intake is associated with a range of adverse outcomes, including iron deficiency anemia, hypertension, preterm delivery, low birth weight, birth defects, intracranial hemorrhage, spontaneous abortion, stillbirth, intrauterine growth retardation, impaired immune function, neural tube defects, visual impairment, labor complications, and even maternal death (2, 6).

Micronutrient malnutrition, particularly deficiencies in iron, iodine, zinc, and vitamin A, is highly prevalent in Ethiopia, ranking 90th out of 116 African countries (7). Validation of the DDS as a key indicator of nutrient adequacy is crucial, as it is the simplest and non-invasive tool to improve the quality of the diet. In addition, only 9% of the households met the DDS across all regions in Ethiopia (8–10). Therefore, we need to validate DDS as a key indicator of nutrient adequacy.

Moreover, empirical evidence suggests that eating the right balance of nutrients and various foods during pregnancy is important to prevent nutritional deficiencies (2, 6). However, while there is a clear link between increasing the diversity of the diet and improving nutrient adequacy, the nature of their association has not yet been sufficiently validated across different cultural settings and documented in developing countries (3, 4). On the other hand, due to differences in socioeconomic conditions and living styles in different developing countries, research validating dietary diversity as a key indicator of nutrient adequacy cannot bring about uniform results. Thus, policy actions that help achieve nutrition and dietary diversity in one context may not yield the same result elsewhere (3).

Even though several studies have assessed the prevalence and the determinants of dietary diversity in Ethiopia, a country characterized by significant variations in dietary consumption practices, none of the studies successfully established a link between DDS and nutrient adequacy (9–13). Therefore, there is a need to get a nuanced understanding of FVS and DDS as key indicators of nutrient adequacy using a simpler and less expensive tool for data collection and analysis than time-consuming and expensive biochemical tests (14). The findings of this context-specific research would have significant implications for designing and implementing country-specific nutrition programs. Therefore, this study aimed to validate FVS and DDS as key indicators of nutrient adequacy in the northern zone of the Sidama region, Ethiopia.

## Materials and methods

### Study setting and study design

A community-based cross-sectional study was conducted from March 1 to 30, 2024, during the dry season following the autumn harvest. The study was conducted in the Boricha and Shebedino districts in the northern zone of the Sidama region. The districts are approximately 300 and 337 kilometers away from Addis Ababa, the capital of Ethiopia, respectively. The districts have 23 and 14 kebeles (lower administrative units) and were chosen at random. A multistage sampling method was applied to select 12 kebeles from the districts. Agriculture is prominent in the districts, serving as the main means of sustenance for 85% of the local community, mainly consisting of grain-, maize-, and haricot bean-producing kebeles (15).

The Shebedino district is located 1,760 to 3,000 m above sea level. The Shebedino district had an estimated total population of 209,063, 48,084 women of reproductive age, and an expected pregnancy rate of 6,272. The other selected district was Boricha, which had an estimated total population of 135,273; the number of women of reproductive age was 31,112, and the expected pregnancy rate was 4,058 (15). The geographical location of the district extends from 6°46’N to 7°01’N and 38°04′E to 38°24′E (16).

### Population and eligibility criteria

The source population for this study consists of pregnant women residing in the northern zone of the Sidama region. On the other hand, pregnant women in the first trimester who were willing to participate in the study were selected as the study population. Only pregnant women who had lived in the study area for at least 6 months were eligible to participate. Pregnant women who were ill, particularly those who were anemic during the data collection period, were excluded from the study since their participation could affect the generalizability of dietary iron intake.

### Sample size determination and sampling technique

Sample size was calculated using OpenEpi version 3.01. The single population proportion formula (N) = {(zα/2)2 p (1-q)}/ (d) 2 was used to compute sample size, where “p” is an attribute that was presented in an estimated proportion (14.7%) of those who practiced diverse diets taken from the previous study (13), 95% confidence interval with 5% margin of error; Zα/2 = 1.96, 14% probability for non-response rate, and a design effect of 1.9. Hence, the total sample size of 424 pregnant women who were in the first trimester was determined for the study.

Twelve kebeles were randomly selected from 37 kebeles in the districts. A systematic random sampling approach was employed to select the study participants from the chosen Kebeles. Eligible women underwent a urine human chorionic gonadotropin (HCG) test to confirm pregnancy, and the gestational age of the pregnancy was confirmed by the principal investigator using menstrual history and clinical examination. The sampling frame was prepared using a list of the selected households containing women with confirmed pregnancies. Based on the size of this population, the calculated sample size was proportionally allocated to each kebele. If multiple eligible women were present in a selected household, one was chosen using a lottery method. If a woman was absent from the home for three consecutive visits during the data collection period, we classified her as a non-respondent.

### Variables of the study

#### Outcome variables

The study consists of primary and secondary outcomes. The primary outcome was nutrient adequacy, whereas DDS and FVS were secondary outcome variables.

#### Independent variables

We categorized the independent variables into individual-level and household-level variables. Individual-level variables were residence, religion, ethnicity, age, school attendance, educational status, occupation, and marital status, which were sociodemographic factors. Pregnancy-related variables were gestational age, parity, and inter-pregnancy interval. Variables related to dietary practices were meal frequency, DDS, and FVS. Household-level variables were family size, residence, mass media usage, agricultural land and livestock ownership, wealth index, and household food security (HHFS).

### Data collection and measurements

We collected the data using a structured and pretested interviewer-administered questionnaire developed based on a review of relevant literature (13, 17, 18). The questionnaire covered sociodemographic and economic characteristics, dietary practice, household family size, food security, sanitation and hygiene, and anthropometric measurement. We calibrated a set of local household utensils and graduated food models before data collection. To maintain its originality and consistency, language experts translated an English version of the questionnaire into “Sidamifa” and then back to English.

The data collection process included four data collectors and two supervisors, who received 4 days of training on the Kobo Toolbox system and 24-h recall interview skills. A pilot test for interactive 24-h recall was conducted on 5% of pregnant women in another kebele of the study. The purpose of the interactive 24-h recall and the details about the data collection arrangements, the details of the 24-h recall interviews, and the procedures used to estimate portion size during training were explained during training. The data collectors conducted face-to-face interviews and recorded the data using the Kobo Toolbox application installed on Android devices. Rigorous supervision included daily examinations and prompt error correction. The collected data were submitted to a central server.

### Assessment of household food security

To estimate overall perceived household food insecurity, we used the Food Insecurity Access Scale questionnaire, comprising nine questions that reflect three different domains of food insecurity, including anxiety and uncertainty about food supply, insufficient food quality, and insufficient food intake. We categorized the households into four levels of food insecurity grades as recommended by the United States Agency for International Development (USAID) Food and Nutrition Technical Assistance III Project (FANTA): food secure, mildly food insecure, moderately food insecure, and severely food insecure (19).

### Assessment of household wealth index

The wealth index was calculated using principal component analysis (PCA) as a combined indicator of life standards based on 17 questions related to ownership of prudently selected household assets (television, mobile phone, radio, and kerosene lamp); housing quality (type and size of house, number of persons per room, type of floor, type of wall, and type of roof material); home facilities (electricity, source of drinking water, cooking fuel, and toilet facility); and means of transport. For each household, the coordinate on the first axis of the correspondence analysis was interpreted as an index of the economic level, and the wealth index was categorized into terciles in subsequent analyses. If the variables were available, we categorized them as “1” and otherwise as “0.”

### Assessment of dietary intake

A multiple-pass qualitative 24-h dietary recall method was adapted to assess the dietary intake of pregnant women, helping overcome the major limitations of recording a single day’s dietary intake using a multiple-pass quantitative 24-h diet recall. The method does not represent a person’s usual intake due to the day-to-day variation of dietary intake (20). Four passes were made to collect detailed information on food items and quantities. In the first pass, the respondents recalled all the foods and drinks consumed in the previous 24 h. The second pass involved a detailed description of food preparation, including cooking methods and the time and place of consumption. In the third pass, portion sizes and amounts of each food and drink consumed were probed using standardized measurement methods. Finally, the entire list of data was reviewed to ensure completeness and accuracy and to identify errors. Respondents reported their food consumption from 25 predefined food groups within 24 h, both at home and outside.

Each food or drink recorded in the household measurement and different calibrated utensils and portion sizes from the 24-h recall was manually converted into weights (in grams). Nutritional values per 100 grams were determined using the Ethiopian Food Composition Table (EFCT) (21). For food items not covered by the EFCT, relevant African countries’ food composition data were utilized, and nutrient values from the USDA table of Nutrient Retention Factors Release 6 (22, 23) were used. The tables were used to calculate the nutrient intake data, which was already processed for cooked foods or ingredients.

### Assessment of food variety score and dietary diversity score

We classified food variety into a predefined list of 25 food groups and further aggregated them into nine food groups. Additionally, we assessed DDS using a 24-h recall questionnaire provided by the FAO. We calculated FVS and DDS by summing up the number and kind of food consumed from the nine groups (24): (1) starchy staple; (2) pulses and legumes; (3) nuts and seeds food groups; (4) dairy food group; (5) fleshy food group; (6) eggs; (7) dark green vegetables; (8) other fruits and vegetables; (9) and other vitamin A vegetables and fruits. Other remaining items, such as tea, sugar, and sweets, were not used in DDS and FVS calculations. The MDD-W was calculated and categorized as per the FAO’s recommendation: “inadequate” for those who consumed < 5 food items and “adequate” for those who consumed ≥ 5 (25). Food group consumption was dichotomized, with a score of 1 assigned if the participant consumed at least five food groups in the past 24 h and 0 otherwise. Women who achieved an FVS and DDS were expected to be more likely to meet their nutrient needs than those who consumed foods from fewer food groups (18).

### Assessment of nutrient adequacy of the diet

To summarize nutrient adequacy, we included those micronutrients having public health relevance (such as vitamin A, thiamin, riboflavin, niacin, vitamin B-6, folate, vitamin B-12, vitamin C, calcium, iron, and zinc) and those related to potential effects on pregnancy outcomes in the study. The distributions of estimated usual intakes of nutrients were compared to the WHO/FAO requirement distributions (23, 26). We calculated an EAR of the nutrient intakes based on the Institute of Medicine (IOM) recommendations (25) when the estimated average requirement (EAR). Usual dietary intake distributions were computed before calculating each nutrient’s nutrient adequacy ratio (NAR). The MAR for a group of food items consumed is equivalent to the prevalence of adequacy for a particular nutrient (27).

The nutrient adequacy of the micronutrients was determined by calculating the nutrient adequacy ratio (NAR, %) of each of the 11 micronutrients for each nutrient. We calculated the intake of each nutrient divided by the recommended intake for that nutrient using WHO/FAO recommended intakes, which are set at two standard deviations above the average requirements (26), which are set at two standard deviations above the average requirements. The calculation was carried out taking into account nutrient requirement distributions and inter- and intra-individual variation in intakes. In the case of iron and zinc, the category for moderate bioavailability was used. As a summary indicator of an overall measure of the nutrient adequacy, the MAR was calculated for the overall diet, where MAR is the sum of each NAR (truncated at 100%) divided by the number of micronutrients (28) as follows: $\text{MAR}=\frac{\sum \text{NAR}}{\text{Number of nutrient}}$.

MAR is equivalent to a population-level estimate of nutrient adequacy. For both NAR and MAR, a value of 100% is ideal since it means that the intake is the same as the requirement. That would mean that the intake of all 11 nutrients, namely vitamin A, vitamin B1, vitamin B2, vitamin B3, vitamin B6, vitamin B9, vitamin B12, vitamin C, calcium, iron, and zinc, is equal to or greater than the RDA, and the requirements for all the nutrients are met. Since no participant had a MAR score of 1 in this study, overall micronutrient intake inadequacy was operationalized to be <0.75 (18, 29).

### Data management and analyses

Data were collected using the Kobo Toolbox system, a free, open-source tool for mobile data collection. Statistical analysis was performed using STATA, statistical software for data science (30). Before doing the main analysis, all necessary variable recoding, computations, and categorizations were conducted. Data were described using frequency distributions, measures of central tendency, and dispersion. For all statistical tests, values of *p* < 0.05 were considered significant. A linear regression model was performed to test statistically significant associations between individual- and household-level determinants and the dietary quality indexes (FVS and DDS). All covariates showing linear association with the indexes with *p*-values less than 0.25 in a univariate model were included in the final model with a *p*-value < 0.05 using adjusted odds ratios (AORs) with 95% confidence intervals (CIs) after controlling for confounding factors. Variables contributing to the variation in the final model were declared as associated factors when the AORs did not contain 1. Potential modifier effects were investigated by including statistical interaction terms in the models to assess the relation between the main explanatory variables and outcome variables.

A multinomial logit model was computed by considering MDD-W as categorical but non-ordered values (31). In this study, we adopted the ordered logit model to estimate the determinants of MDD-W since the dependent variable has an ordered nature—adequate and inadequate dietary diversity about age differences and socioeconomic variability among urban and rural areas. Pearson’s correlation tests were conducted to determine significant relationships between MDD-W and MAR and nutrient adequacy of individual nutrients and between FVS and MAR and nutrient adequacy of individual nutrients. The household wealth index was constructed using PCA as a combined indicator of life standards. Factor analysis was used to analyze food insecurity scale indicators.

ROC curve analysis was computed to provide a graphical representation of the range of possible cutoff points with their associated sensitivity vs. 1-specificity (i.e., false positive rate) and to assess the accuracy of DDS and FVS to classify pregnant women with a low or high MAR. Sensitivity indicates the proportion of pregnant women with higher MAR values, while specificity indicates the proportion of pregnant women with a lower MAR. (32) The area under the curve (AUC) was calculated using a ROC curve based on the nutrient adequacy as either yes (MAR ≥ 0.387) or no (MAR < 0.387). Additionally, further analyses were conducted to assess the performance of the MDD-W at different MAR thresholds, which were set between 0.50 and 0.85. The AUC values were then interpreted according to predefined criteria, which categorized them as fail (0.5–0.6), poor (0.6–0.7), fair (0.7–0.8), good (0.8–0.9), or excellent accuracy (0.9–1.0) (32). We considered an AUC cutoff ≥0.70 as a rule-of-thumb criterion to indicate acceptable predictive power for DDS and FVS. The optimal cutoff points were identified by selecting the points that maximized the Youden J statistic (sensitivity + specificity − 1) (the larger the better) (33). A *p*-value less than 0.05 was considered statistically significant.

## Results

### Socio-demographic and economic characteristics of the respondents

All the study participants had participated in the study. The mean and standard deviation (SD) age of the participants was 26.29 (±5.62) years. More than three-fourths (78.8%) of the respondents could read and write. The family size of the participants ranged from 2 to 6 people. 407 (96%) were Protestant Christians, 420 (99%) were Sidama in ethnicity, and 418 (98.1%) were married. 91.5% of the respondents were in the poor wealth quintile. Nearly 90% of the households were food insecure, with a third (36.1%), and 99% of the respondents could not afford their food and could not establish an extra mealtime. More than half (52.8%) of the respondents had their meal 1–2 times per 24 h, and 34.2% skipped a meal at least three times a week (Table 1).

**Table 1:** Socio-demographic and economic characteristics of pregnant women in Northern zone of the sidama region, Ethiopia, 2024.

Variables	Categories of variables	Frequency	Percent
Age	≤20	142	33.5
	>20	282	66.5
Marital status	Married	418	98.6
	Divorced	6	1.4
Maternal education	Literate	334	78.8
	Illiterate	90	21.2
Level of education	Primary	323	76.2
	Secondary and above	101	23.8
Residence	Rural	312	73.6
	Urban	112	26.4
Land ownership	Yes	354	83.5
	No	70	16.5
Family number	2–4	105	24.8
	≥5	319	75.2
Religion	Protestant	407	96.0
	Others ^a^	17	4.0
Ethnicity	Sidama	420	99.0
	Others ^b^	4	1.0
Women’s occupation	Housewife	372	87.7
	Employed/merchant	52	12.3
Spouse occupation	Farmer	352	83.0
	Employed /merchant	72	17.0
Wealth index	Rich	36	8.5
	Poor	388	91.5
Can afford for food	Yes	161	38
	No	263	62.0
HHFS	Secured	61	14.4
	In-secured	363	85.6

### Food variety and dietary diversity of the respondents

**Figure 1:**
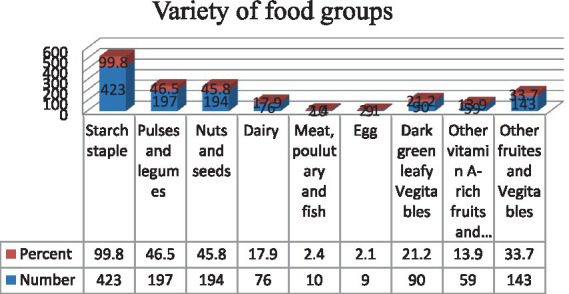
Proportion of pregnant women consuming varieties of food groups in the Northern Sidama Zone, Ethiopia (*n* = 424).

The prevalence of DDS (≥5 food groups) and FVS was 39.9% (95% CI: 35.6, 44.3) and 36.3% (95% CI: 31.6, 41.0), respectively. The range of food groups consumed varies from a minimum of one to a maximum of seven. The median DDS was 3.0 ± 1.48. On average, 3.42 and 4.67 pregnant women met the DDS and FVS, respectively, in the last 24 h prior to data collection. The vast majority of them consumed starchy staple foods, followed by dark leafy vegetables (79.2%). Nutrition-dense food commodities such as flesh food (2.1%), eggs (0.03%), other fruits and vegetables (13.9%), and milk and milk products (dairy) (18.4%) were the least consumed food groups (Figure 1).

The overall prevalence of adequate micronutrient intake, defined as MAR ≥ 0.50, was 20.8% (95% CI: 16.7, 24.8). This prevalence was calculated among the 10 micronutrients after excluding B3, which was adequate for all participants.

**Figure 2:**
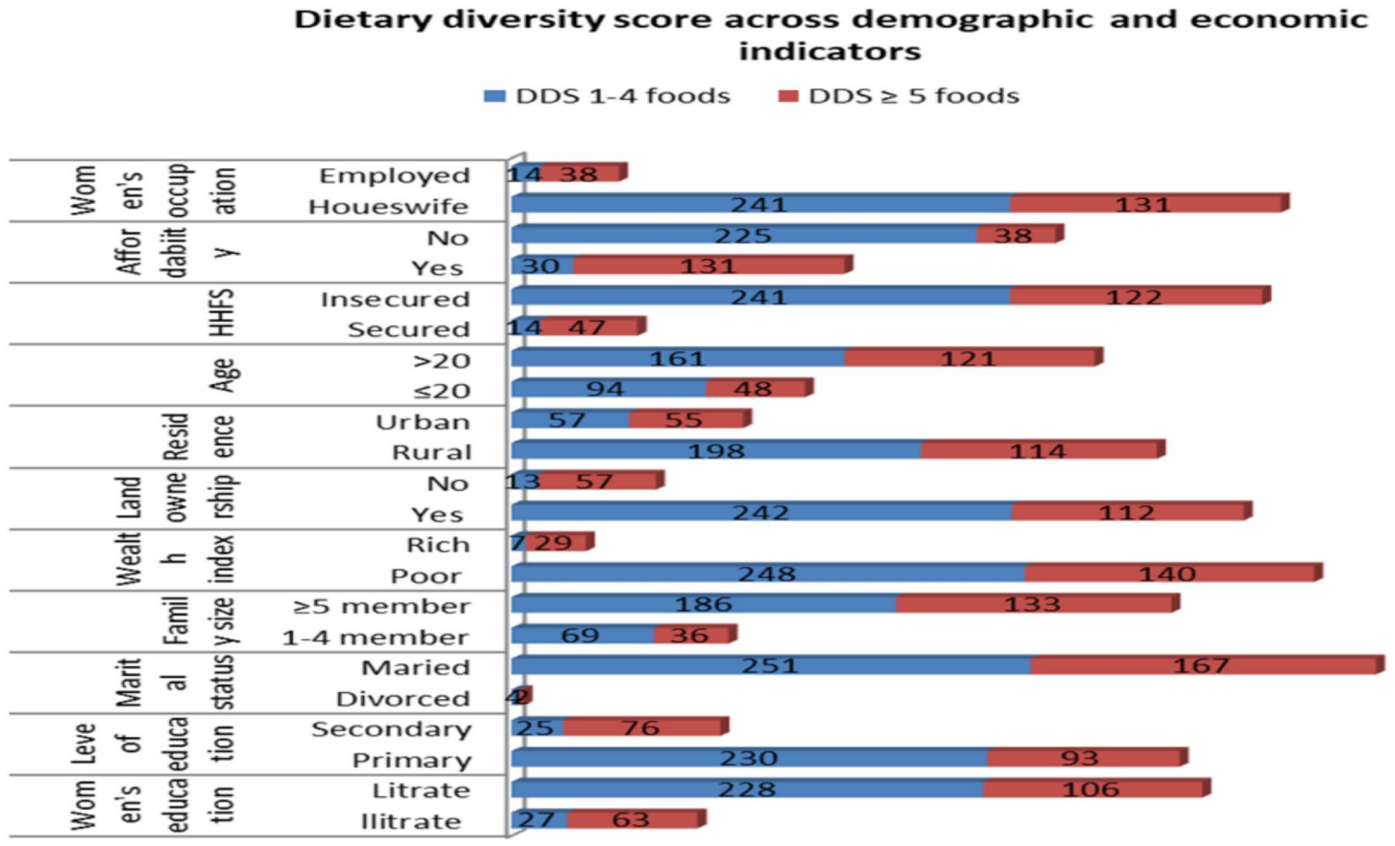
Dietary diversity scores against demographic and economic indicators among pregnant women in the northern zone of the Sidama region, 2024 (*n* = 424).

Nearly all (98.1%) respondents had visited health facilities for the first antenatal care (ANC), and >90% had received counseling on women’s dietary diversity. Among the study participants who consumed an adequate diet, 378 (89.2%) were in the age group of ≥46 years, 41.6% of them were urban residents, and 68.9% of them were from a high-wealth index. On the other hand, among the study participants who consumed an inadequate diet, 53.1% of them could not afford their food, 58.5% of them were from a poor wealth index, and 56.8% of them were housewives, respectively (Figure 2).

**Figure 3:**
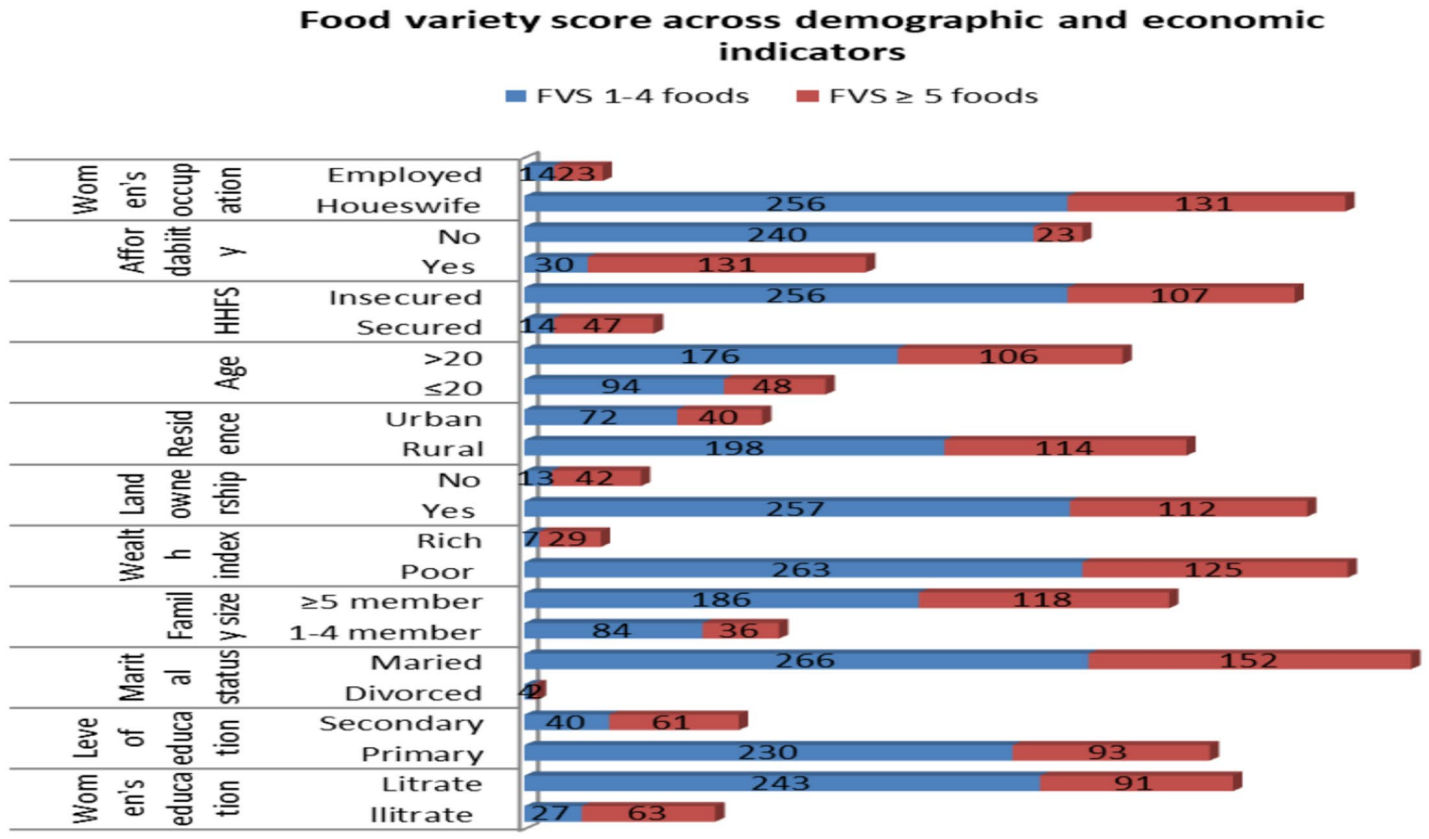
Food variety scores against demographic and economic indicators among pregnant women in the northern zone of the Sidama region, 2024 (*n* = 424).

Similarly, among the study participants with low food variety, 62.03% were housewives, 56.6% could not afford food, 68.4% had a family size of ≥5, and 43.9% were from a poor wealth index, respectively (Figure 3).

### Dietary intakes and nutrient adequacy of the respondents

The study demonstrates that median intakes of micronutrients fall significantly below the estimated average requirements (EARs) for the majority of the 11 micronutrients evaluated. The estimated intake prevalence is alarmingly low, ranging from 3 to 25% for vitamin C, calcium, vitamin B12, and zinc. It remains low (27–42.1%) for vitamin A, thiamin, vitamin B6, and folate; moderate (51–58%) for iron and riboflavin; and notably high (100%) for niacin. Between 84 and 100% of respondents consumed each of the nutrients in amounts below the EAR. Every single respondent had an intake that failed to meet the EAR for vitamin A, thiamine, folate, vitamin B12, and vitamin C. Moreover, 84 to 97.2% of respondents were below the estimated average requirement for niacin, riboflavin, vitamin B6, zinc, calcium, and iron.

The mean of MAR for 11 micronutrients was very low (0.387). The mean of the individual nutrients ranged from 0.03 to 32.27 for the micronutrients (Table 2). More than three-fourths of the study subjects had a MAR of less than 50%, whereas 22% had a MAR of 50–71%. There was no difference between the urban and rural residents (*p*-value = 0.265); however, a significant difference was noticed in dietary diversity (*p*-value = 0.001) and food variety (*p*-value = 0.001) between urban and rural residents.

**Table 2:** Intake of nutrients together with safe level of intake and the nutrient adequacy ratio among pregnant women in Northern sidama zone, Ethiopia (*n* = 424).

Nutrients	Intake/person/day				RDA in kcal	Nutrient adequacy ratio (NAR)	
	Median	Q25	Q75	% of median intake	Mean ± SD	Mean ± SD	% of <RDA/EAR
Calcium, mg	93.70	73.80	141.30	9.9	950 mg	0.10 (0.06)	94.6%
Iron	13.20	12.40	14.70	50.8	26 mg^2^	0.22 (0.08)	94.8%
Zinc, mg	3.30	2.90	3.60	29.2	11.3 mg^2^	0.31 (0.08)	91.5%
Vitamin A μg	1,210	1,190	1,260	26.9	4,500^d^ μg	32.27 (10.94)	100%
Thiamine mg,	0.03	0.02	0.04	30	0.1 mg/MJ	0.33 (0.15)	100%
Riboflavin, mg	1.10	1.10	1.20	57.9	1.9 mg	0.56 (0.26)	89.2
Niacin, mg	1.60	1.10	2.10	100	1.6 NE/MJ	1.06 (0.48)	84.2%
Vitamin B_6_ mg,	0.80	0.70	0.90	42.1	1.9 mg	0.45 (0.13)	97.2%
Folate, μg	185.80	174.80	274.80	31	600 μg	0.35 (0.12)	100%
Vitamin B_12_ μg	1.00	0.70	1.60	22.2	4.5 μg	0.24 (0.13)	100%
Vitamin-C, mg	2.60	1.60	19.90	2.5	105 mg	0.10 (0.08)	100%

^a^Average requirement of energy was calculated multiplying estimates of resting energy expenditure (REE). ^b^Recommended daily allowance for carbohydrate and fat was calculate based on the intervals of E% with the sum of 100%. ^c^Recommended daily allowance for protein was calculate based on 0.83 g/kg body weight per day for adults. ^d^1RE = 6 μg of dietary β-carotene.

### Validation of MDD-W in predicting nutrient adequacy of the respondents

We determined a cutoff point of MAR to test the predictive power of a test that indicates the accuracy of the test to discriminate between adequate and inadequate diets. In the ROC curve analyses, 100% is ideally an acceptable value (32). However, no woman in our sample reached this value; we therefore used 70% of MAR as a cutoff point value for nutrient adequacy. In the analysis, DDs exhibited good predictive ability for the adequacy of 11 micronutrients at a 70% cutoff point in MAR ≥ 0.378 (AUC = 0.839, 95% CI: 0.80 to 0.88) with the Youden J index of 0.06. Thus, DDS in this analysis was found to be an indicator of the nutrient adequacy, with a sensitivity of 69.9% and a specificity of 5%. This corresponded to a very good predictive quality and showed strong diagnostic performance and indicated a high (84%) ability to identify those respondents with an adequate dietary diversity, significant at a *p*-value of <0.05.

**Figure 4:**
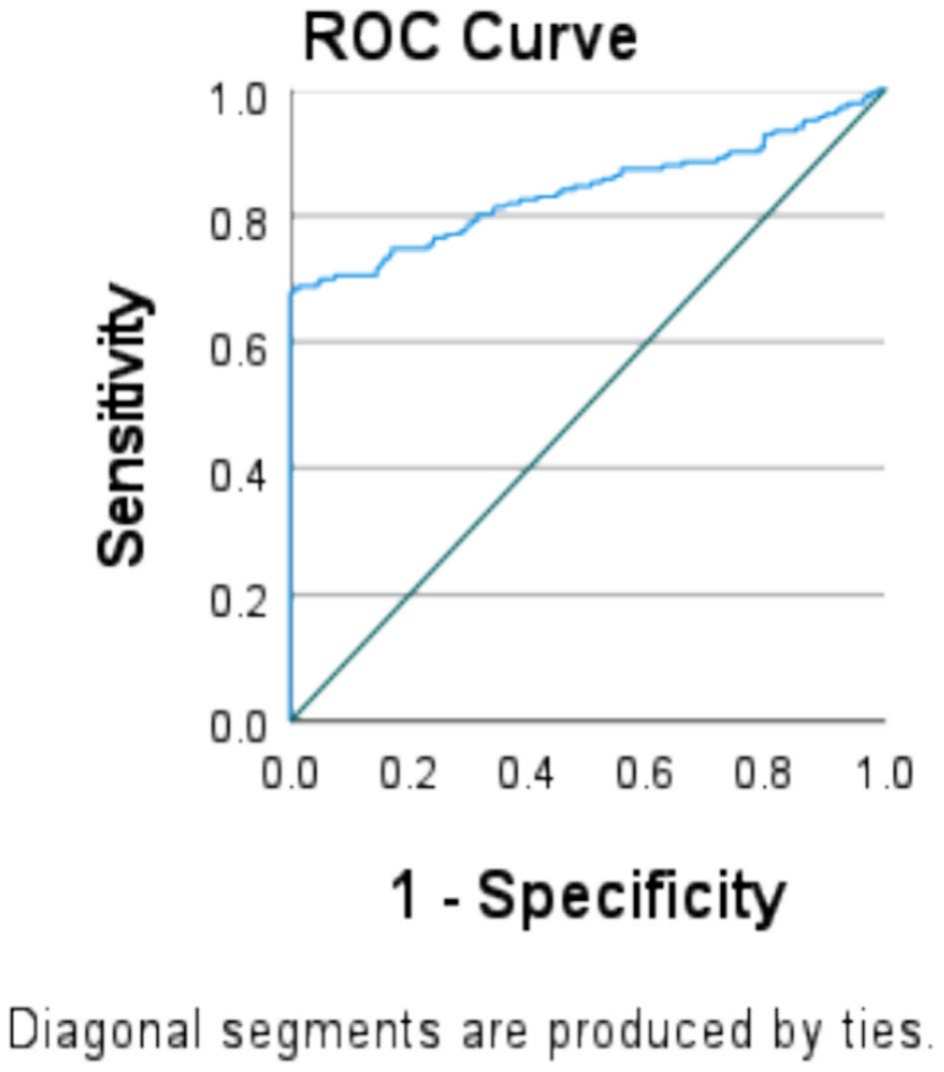
Dietary diversity score indicates MAR of the micronutrients at 3.87 in the ROC curve model at the cutoffs of 70% sensitivity and specificity among pregnant women in the northern zone of the Sidama region (*n* = 424).

The proportions of pregnant women with a higher and lower MAR were indicated by sensitivity and specificity. It was correctly identified using the cutoff point of 70% for each score of the ROC curve (Figure 4).

Furthermore, the most aggregated diversity indicators of the nine food groups were not the best predictors of individual NAR. At least with the 70% cutoff at area under the curve (AUC), AUC = 62.5 for calcium, AUC = 62.6 for iron, AUC = 59.9 for zinc, AUC = 65.4 for vitamin B6, AUC = 53.5 for vitamin B9, and AUC = 68.8 for vitamin C. These correspond to poor quality of predictions and have shown poor diagnostic performances at *p* < 0.05. The model indicated a < 70% chance of correctly ranking a random positive value higher than a random negative value of the nutrient adequacy (Table 3).

**Table 3:** Area under the curve (AUC) of the mean adequacy ratios (MAR) and dietary diversity score among pregnant women in the Northern sidama zone (*n* = 424).

Nutrient	Optimal cutoffs	AUC	*p* value	Sensitivity and specificity at optimal cutoffs		95% CI of AUC	
				Sensitivity	1-Specificity	Lower limit	Upper limit
MAR (DDS)	3.87	0.839	0.000	69.9%	0.05%	0.80	0.88
MAR (FVS)	3.82	0.709	0.001	86.5%	0.50%	3.49	4.03
Calcium	0.10	0.625	0.000	46.2%	37.3%	0.57	0.68
Iron	0.22	0.626	0.000	56.2%	34.1%	0.57	0.68
Zinc	0.31	0.599	0.001	34.3%	19.6%	0.54	0.66
Vit B_6_	0.45	0.654	0.000	57.4%	35.3%	0.60	0.71
Vit B_9_	0.01	0.535	0.017	57.4%	35.3%	0.48	0.58
Vitamin C	0.10	0.688	0.000	46.2%	37.3%	0.63	0.74

### Validation of FVS in predicting nutrient adequacy of the respondents

On the other hand, the most aggregated food variety indicator of nine food groups remained a poor predictor of 11 micronutrients at MAR ≥ 0.378 (AUC = 0.709, 95% CI: 3.49 to 4.03) with the Youden J index of 0.36. This corresponded to a poor quality of prediction, which showed a poor diagnostic performance. There was a lower (71%) ability to identify the respondents with an adequate and inadequate diet (a *p*-value of <0.05). Thus, FVS was a poor indicator of nutrient adequacy with a sensitivity of 87% and a specificity of 50% (Table 3).

### Correlation of MAR with DDS and FVS

A few food groups, like dark green leafy vegetables and legumes, were significantly correlated with MAR. Other groups—some of which were known to be nutrient-dense—were also significantly correlated with MAR (e.g., dairy and eggs). Starchy staples, which provided the majority of micronutrient intakes, were not correlated with MAR. Both the DDS and FVS were significantly correlated with each other (*ρ* = 0.748; 95% CI: 0.702 to 0.787), and the DDS was positively correlated with the MAR of micronutrients (*ρ* = 0.159, 95% CI: 0.065–0.250) (*p*-value = 0.001). DDS was positively correlated with the NAR of calcium (r = 2.88, 95% CI: 0.198–0.373), zinc (*ρ* = 0.268, 95% CI: 0.177–0.354), vitamin B6 (*ρ* = 0.108, 95% CI: 0.013–0.201), and vitamin B12 (*ρ* = 0.166, 95% CI: 0.178–0.372). The variations in NAR of calcium (28.8%), zinc (26.8%), vitamin B6 (10.8%), and vitamin B12 (16.6%) during pregnancy were accounted for by maternal dietary diversity.

On the other hand, DDS was negatively correlated with NAR of vitamin A (*ρ* = −0.106, 95% CI: −0.119-0.010). The variation in the adequacy of the nutrient (10.6%) was negatively affected by dietary diversity. Additionally, the DDS was not significantly correlated with the NAR of the micronutrients. Iron (*ρ* = 0.069, 95% CI: −0.026 to 0.164), thiamin (*ρ* = 0.047, 95% CI: −0.049 to 0.141), riboflavin (*ρ* = 0.001, 95% CI: −0.094 to 0.096), niacin (*ρ* = 0.049, 95% CI: −0.46 to 0.144), folate (*ρ* = −0.012, 95% CI: −0.107 to 0.083), and vitamin C (*ρ* = −0.046, 95% CI: −0.140 to 0.050) were among the nutrients not correlated significantly. However, FVS was positively correlated with NAR of vitamin B12 (*ρ* = 0.137, 95% CI: 0.042–0.229), in which the variation in the adequacy of the nutrient (10.6%) was accounted for by dietary diversity. The FVS was negatively correlated with the NAR of calcium (*ρ* = −0.120, 95% CI: −0.213 to −0.025), in which the variation in NAR of calcium (12.0%) was negatively affected by maternal dietary diversity (Table 4).

**Table 4:** Pearson’s correlation coefficient (*r*) between nutrient adequacy ratio (NAR), mean adequacy ratio; and dietary diversity score and food variety score among pregnant women in the Northern sidama zone, Ethiopia (*n* = 424).

MAR or NAR	Correlation coefficient (r)	*p* value	95% CI	
			Lower limit	Upper limit
Maternal dietary diversity score				
MAR (micronutrient)^**^	0.159	0.001	0.065	0.0250
Calcium^**^	0.288	0.000	0.198	0.373
Zinc^**^	0.268	0.001	0.177	0.354
Beta-carotene^*^	−0.106	0.030	−0.199	−0.010
Vit-B_6_^**^	0.108	0.026	0.130	0.201
Vit B_12_^**^	0.166	0.001	0.178	0.372
Iron	0.069	0.153	−0.026	0.164
Thiamin	0.047	0.338	−0.049	0.141
Riboflavin	0.001	0.987	−0.094	0.096
Niacin	0.049	0.311	−0.46	0.144
Folate	−0.012	0.803	−0.107	−0.083
Vitamin C	−0.046	0.349	−0.140	0.050
Maternal food variety score				
Vitamin B_12_^**^	0.137	0.005	0.042	0.229
Calcium^*^	−0.120	0.013	−0.213	−0.025

** Positively correlated with maternal dietary diversity and food variety scores.

* Negatively correlated with maternal dietary diversity and food variety score.

### Factors associated with DDS, FVS, and MAR

In a multivariate linear regression analysis, each model explained 33.6, 21.4, and 15.1% of the variation in DDS, FVS, and MAR, respectively. The study indicated that a high level of food security (AOR = 12.60, 95% CI: 5.20 to 30.00), a rich wealth index (AOR = 0.30, 95% CI: 0.10 to 0.80), a family member of 2–4 (AOR = 2.71, 95% CI: 1.91 to 3.83), school attendance (AOR = 3.30, 95% CI: 1.80 to 5.90), high-level education (AOR = 2.20, 95% CI: 1.60 to 3.10), and employment (AOR = 2.30, 95% CI: 1.40 to 3.90) were found to be positive predictors of DDS compared to their counterparts. On the other hand, school attendance (AOR = 2.30, 95% CI: 1.20 to 4.30) and having a family size of 2–4 (AOR = 3.10, 95% CI: 2.10 to 4.30) were found to be positive predictors of women’s FVS compared to their counterparts.

Furthermore, ownership of agricultural land (AOR = 1.41, 95% CI: 0.86 to 1.42) was a positive predictor of the MAR (Table 5). Women aged ≤20 years (AOR = 3.17, 1.11 to 9.04) were 3.17 times more likely to have an adequate DDS compared to those who were ≥20 years old. There was a significant difference in the means of sociodemographic and economic determinants of dietary diversity score, such as wealth index (*p*-value = 0.059), food insecurity scale (*p*-value = 0.001), monthly income of the households (*p*-value = 0.001), occupation of the respondents (*p*-value = 0.001), birth interval (*p* = 0.047), and meal frequency (0.039) between urban and rural residents.

**Table 5:** The linear regression modeling of predictors of dietary diversity, food variety and mean adequacy among pregnant women in northern sidama zone, Ethiopia, 2024 (*n* = 424).

Variables		Crude odds ratio (COR)		Adjusted odds ratio (AOR)	
		*p* value	(95% CI)	*p* value	(95% CI)
Dietary diversity score (DDS)					
School attendance	Yes	0.001	7.50 (4.00 to 14.20)	0.001	3.30 (1.80 to 5.90)
	No	Ref			
Level of education	≤Primary school	Ref			
	≥Secondary school	0.001	3.50 (2.60 to 4.80)	0.001	2.20 (1.60 to 3.12)
Occupation	House wife	Ref			
	Employed	0.001	4.40 (2.50 to 7.80)	0.002	2.30 (1.40 to 3.90)
Food insecurity scale	Food secured	0.001	4.90 (3.21 to 7.40)	0.001	12.60 (5.20 to 30.00)
	Food in-secured	Ref			
Family size	2–4 households	0.001	4.50 (3.320 to 6.01)	0.001	2.71 (1.91 to 3.83)
	≥5 households	Ref			
Food variety score (FVS)					
School attendance	Yes	0.001	5.20 (11.70 to 42.1)	0.014	2.30 (1.20 to 4.30)
	No	Ref			
Family size	2–4 households	0.001	4.12 (3.01 to 5.61)	0.001	3.10 (2.10 to 4.30)
	≥5 households	Ref			
Mean adequacy ratio					
Ownership of agricultural land	≤Half hectare	Ref			
	>Half hectare	<0.001	1.13 (0.86 to 1.40)	<0.001	1.41 (0.86 to 1.42)
Source of food consumed	Purchasing	<0.001	−0.43 (−0.64 to −0.21)	<0.001	−0.42 (−0.64 to −0.20)
	Production	Ref			

## Discussion

To maintain a healthy pregnancy, a woman needs a balanced diet of protein, fruits, vegetables, and whole grains (34). Nonetheless, the finding highlighted that the majority of women consumed starchy staples with little variety, contributing to the burden of micronutrient deficiencies (26). This was due to widespread food insecurity among pregnant women, particularly those from households in the lowest wealth quintile. Consistent with this, reports from LMICs and FAO highlighted that monotonous diets based mainly on grains, roots, and tubers are common in areas of high food insecurity that contribute to the burden of malnutrition (3, 26). Therefore, intake of various foods and a diversified diet during pregnancy may ensure adequate essential nutrients (26, 31) and promote good health (35, 36).

This study reported lower DDS and FVS than the benchmark of MDD-W. The finding was similar to reports from the studies conducted in South Africa and Mali (37, 38). Nonetheless, a report from rural Mali indicated a higher mean food variety score and a slightly elevated DDS than the benchmark (37). The discrepancy might be due partly to the study areas. Furthermore, the intake of an adequate and diverse diet and variety of food was low in the study area. Inconsistent with this, some studies in Ethiopia (36, 37) and in other developing countries (39–42) reported a slightly higher intake of adequate, diverse diets.

The possible reasons for the discrepancy might be economic inflation impacting food expenditures (43). Variations in sociodemographic characteristics among study participants in Ethiopia and other developing countries may create a difference in having diverse diets. Thus, the studies recommended that the government strengthen women’s empowerment, rights, access to education, and economic opportunities to ensure nutrient adequacy and quality of the diet during pregnancy (44, 45). The current study supported the recommendation, in which the finding was significantly associated with food security, wealth index, and women’s education.

Dietary diversity has been identified as a potentially useful candidate and used as an indicator of dietary quality (3, 4). However, a report from rural Mali highlighted that DDS or FVS do not give a full picture of the nutrient adequacy of the diet. The report also indicated that DDS or FVS does not tell which food groups contribute most to the dietary quality (28). This is because the diets of individuals in developing countries may show less day-to-day variation in diversity. The report was supported by the findings from a rural area of Burkina Faso, where the mean DDS of women was increased by 0.7 food groups (3). In line with this, the present analysis indicated that the proportion of pregnant women with a nutrient intake below the recommendations varied between the kinds and intakes of the nutrients.

The finding indicated that the nutrient intakes were inadequate and far below the estimated average requirement (3, 46). Notably, inadequate intakes were noticed for all micronutrients except for niacin and other micronutrients of public health importance (i.e., zinc and vitamin A) and calcium (46). The finding was similar to that reported from the study in a low-resource setting (3), where the median intakes of riboflavin, niacin, vitamin B12, folate, and vitamin A were below the estimated average requirements. Additionally, reports from Mali indicated that calcium intake was below adequate (37). Nonetheless, the finding was inconsistent with what was reported from China (47), where the MAR was moderate and significantly higher in participants with a diverse diet. The discrepancy might be due to socioeconomic variations among study participants.

Research evaluating maternal and child undernutrition highlighted an insufficiency of multiple micronutrients, including zinc, folic acid, calcium, and vitamin D (48). Other research recognized that deficiencies of multiple micronutrients were more common than one in isolation (49). Consistent with this, the present study indicated a wide variation in nutrient adequacy for individual micronutrients, ranging from 0.03 to 32.27. The estimated prevalence of nutrient adequacy was the lowest (0.1) for calcium and vitamin C, ranged from 0.22 to 0.56 for iron, vitamin B12, zinc, thiamin, folate, riboflavin, and vitamin B6, and was 1.06 for niacin and 32.27 for vitamin A.

Inconsistent with this, a report from Mali estimated that the prevalence of nutrient adequacy was highest for zinc, vitamin C, and vitamin B6. The possible reason for the discrepancy was the difference in the study area, in which the research was conducted among urban women who consumed animal-source foods (ASF) and millet, staple diets that are good sources of zinc and iron (37). However, the estimated prevalence for folate, vitamin B12, riboflavin, and calcium was similarly the lowest in the study from Mali, as found in this analysis.

Furthermore, the prevalence of calcium and vitamin C NAR was very low. The finding was not similar to that reported from the urban sample of women in Mali (37), where the highest prevalence of vitamin C adequacy was seen. A possible reason for the discrepancy might be poor intake of ASF and citrus fruits, such as oranges, lemons, and strawberries. The nutrient-dense food group was correlated with the MAR of the micronutrients (2, 48), providing a summary of the information and underscoring the quality of women’s diets (2). Reports from low-resource settings indicated that the DDS of pregnant women has a positive correlation with MAR. The report ranged from 35% in Bangladesh, 38% in Burkina Faso, 47% in Mali, 47% in Mozambique, and 32% in the Philippines (3). The current study supports these findings. Nonetheless, a higher MAR of the 11 micronutrients was reported from Iran (371.07) (49), 63% from South Africa (38), and 0.161 to 0.484 from China (47). The possible reason for the discrepancy was differences in socioeconomic status between the study subjects. The majority of the study population of this analysis was from rural areas where intake of nutrient-dense food, particularly animal-source food, was significantly lower than the RDA.

DDS and FVS were significantly correlated with each other. The DDS was positively correlated with the MAR of the micronutrients. The finding was consistent with this: there was a strong, significantly positive relationship between the NAR of respective micronutrients and the MAR with the DDS in the study conducted in South Africa (18). However, except for calcium, zinc, and vitamin B6, most individual nutrient intakes did not correlate with FVS and DDS indicators. This is inconsistent with reports from Burkina Faso (3), where, except for folate, iron, and zinc, most individual micronutrient intakes were positively correlated with the DDS.

Similarly, the DDS constructed for this analysis had a positive and significant correlation with the composite indicator of the micronutrient adequacy ratio of some individual micronutrients, such as calcium, zinc, vitamin B6, and vitamin B12. This illustrates the potential of a simple score of DD for use as an indicator of the micronutrient adequacy of the diet. These findings were similar to the report from rural Bangladesh and Burkina Faso (3). In both countries, dietary diversity indicators were correlated with the MAR of all 11 micronutrient adequacies. Nonetheless, we found a significantly negative relationship between vitamin A and DDS. The finding was in line with the findings reported from India and South Africa (18, 50).

The risk factors for inadequate dietary intake and nutrient deficiency during pregnancy are multifactorial (51). The relative contribution of each of these factors varied greatly based on socioeconomic characteristics, such as wealth index, maternal occupation, education level, employment status, monthly income, and household food security status (45, 51); geographical location/residence (10, 52); seasonal variations (53–55); and dietary practice (53). Pregnant women aged ≥46 years were more likely to have adequate dietary diversity compared to those aged 18–45 years, consistent with a local study conducted in southwest Ethiopia (12). Similarly, the current study found that respondents from urban areas and wealthier households had higher dietary diversity scores. The finding aligns with those reported in urban Mali and Burkina Faso (3, 37).

The current study reported that 33% of the rural and 55% of the urban respondents met the MDD-W. Inconsistent with this, the maximum DDS was achieved, according to reports from Sri Lanka (53), Burkina Faso (3), and South Africa (18). The discrepancy might be that the current study reported high food insecurity in rural areas rather than urban areas. This, in turn, was due to the agro-ecological, sociodemographic, soil and water conservation, and land cultivation of the society. These conditions could affect food security more in rural than urban areas, where livelihoods mostly depend on trade and employment (56). Likewise, pregnant women from households having a monthly income of less than 1,000 ETB had an inadequate DDS compared to those having a monthly income of > 1,000 ETB. The finding was in line with some local studies (10, 57).

Consistent with this, primary education had a significant association with inadequate dietary diversity compared to those who completed high school or higher in this analysis. This finding was in line with the findings reported from studies conducted in northwest Ethiopia (58), Addis Ababa (45), and other developing countries (3). In addition, only 7% of unemployed women had met an MDD. The finding was in line with studies conducted in Nekemite town (51), northwest Ethiopia (58), Alamata General Hospital (57), and Dire Dawa city (59). Furthermore, pregnant women who had a meal frequency of three or more times a day had achieved adequate dietary diversity. This was because a higher meal frequency increases the likelihood of various food items being taken in within 24 h (60). Reports from studies conducted in different parts of Ethiopia (10, 57, 58) confirmed that pregnant women who consumed more than 3 times within 24 h and established extra meal times were more likely to achieve adequate dietary diversity.

## Conclusion

Inadequate dietary intake and nutrient deficiency during pregnancy varied with the socioeconomic characteristics of the study participants. MDD-W had a positive correlation and a good predictive ability in determining micronutrient intake adequacy and identifying the respondents having adequate and inadequate DDS. The sensitivity and specificity of the DDS in the ≥5 food group’s standard cutoff were 69.9 and 5.3%, respectively. However, FVS was found to be a poor predictor of nutrient adequacy.

## Recommendation

The Ethiopian government should improve women’s rights, access to education, and economic opportunities to enhance maternal nutrition programs and increase nutrient adequacy. Previous studies have shown varied findings that also differ from FAO recommendations regarding the standards and cutoffs for assessing WDD-W and its relation to micronutrient adequacy, warranting further investigation.

## Limitations of the study

The findings may not be generalizable to other low-income settings because the study was conducted in a specific cultural setting in Ethiopia. Additionally, the study excluded anemic women, which could affect the generalizability of the findings concerning women with anemia. We used the 24-h recall method to assess the women’s DDS, although it is susceptible to recall biases and day-to-day variability. Careful design of the initial 24-h recall protocol and standardized multiple-pass interviewing techniques were used to minimize errors generated by memory lapses and distortions. Additionally, memory aids such as photographs and “probing” have been used to identify the correct species.

## Data Availability

The raw data supporting the conclusion of the article will be made available by the authors without undue reservation.

## Glossary

ASF: Animal source food
ANC: Ante-natal-care
AOR: Adjusted odds ratio
AUC: Area under curve
CI: Confidence interval
COR: Crude odds ratio
DDS: Dietary diversity score
ETB: Ethiopian birr
EFCT: Ethiopian food composition table
EAR: Estimated average requirement
FAO: Food and Agriculture Organization
FANTA: Food and nutrition technical assistance
FVS: Food variety score
HFIAS: Household food insecurity access scale
HHFS: House Hold food security
HCG: Human chorionic gonadotropin
IOM: Institution of Medicine
IRB: Institution Review Board
LMICs: Low and middle income countries
MAR: Mean adequacy ratio
MDD: Minimum dietary diversity
MMD-W: Minimum dietary diversity of women
NAR: Nutrient adequacy ratio
NORAD: Norwegian Agency for Development Cooperation
PCA: Principal component analysis
RDA: Recommended daily allowance
REE: Resting energy expenditure
RNI: Recommended nutrient intakes
ROC: Receiver operating character
SD: Standard deviation
USAID: United States Agency for International Development
WHO: World Health Organization
